# Inoculation with Plant Growth-Promoting Bacteria to Reduce Phosphate Fertilization Requirement and Enhance Technological Quality and Yield of Sugarcane

**DOI:** 10.3390/microorganisms10010192

**Published:** 2022-01-17

**Authors:** Poliana Aparecida Leonel Rosa, Fernando Shintate Galindo, Carlos Eduardo da Silva Oliveira, Arshad Jalal, Emariane Satin Mortinho, Guilherme Carlos Fernandes, Evelyn Maria Rocha Marega, Salatiér Buzetti, Marcelo Carvalho Minhoto Teixeira Filho

**Affiliations:** 1 Department of Plant Health, Rural Engineering, and Soils, São Paulo State University, Ilha Solteira 15345-000, Brazil; polianaleonelrosa@gmail.com (P.A.L.R.); ces.oliveira@unesp.br (C.E.d.S.O.); arshad.jalal@unesp.br (A.J.); emariane.satin@unesp.br (E.S.M.); guilherme.carlos.fernandes@gmail.com (G.C.F.); evelynmarega8@gmail.com (E.M.R.M.); sbuzetti@agr.feis.unesp.br (S.B.); mcmtf@yahoo.com.br (M.C.M.T.F.); 2 Center for Nuclear Energy in Agriculture, University of São Paulo, Piracicaba 13416-000, Brazil

**Keywords:** phosphorus, inoculation, plant-cane, reduction of phosphate fertilization, plant growth promotion, *Saccharum* spp.

## Abstract

Phosphorus (P) is a critical nutrient for high sugarcane yields throughout its cultivation cycles, however, a higher amount of P becomes rapidly unavailable to plants due to its adsorption to soil colloids. Some plant growth-promoting bacteria (PGPBs) may be able to enhance P availability to plants and produce phytohormones that contribute to crop development, quality, and yield. Thus, this study aimed to evaluate leaf concentrations of nitrogen (N) and P, yield, and technological quality of sugarcane as a function of different levels of phosphate fertilization associated with inoculation of PGPBs. The experiment was carried out at Ilha Solteira, São Paulo—Brazil. The experimental design was randomized blocks with three replications, consisting of five phosphorus rates (0, 25, 50, 75, and 100% of the recommended P_2_O_5_ rate) and eight inoculations, involving three species of PGPBs (*Azospirillum brasilense*, *Bacillus subtilis,* and *Pseudomonas fluorescens*) which were applied combined or in a single application into the planting furrow of RB92579 sugarcane variety. The inoculation of *B. subtilis* and *P. fluorescens* provided a higher concentration of leaf P in sugarcane. The P_2_O_5_ rates combined with inoculation of bacteria alter technological variables and stalk yield of sugarcane. The excess and lack of phosphate fertilizer is harmful to sugarcane cultivation, regardless of the use of growth-promoting bacteria. We recommend the inoculation with *A. brasilense* + *B. subtilis* associated with 45 kg ha^−1^ of P_2_O_5_ aiming at greater stalk yield. This treatment also increases sugar yield, resulting in a savings of 75% of the recommended P_2_O_5_ rate, thus being a more efficient and sustainable alternative for reducing sugarcane crop production costs.

## 1. Introduction

Sugarcane (*Saccharum officinarum* L.) is one of the world’s main agricultural crops, and its largest producing country is Brazil, which accounts for 34% of global production [1]. In the 2018/19 crop season, national production reached 620.4 million tons, occupying an area of 8.6 million hectares with an average yield of 72.2 t ha^−1^, where the state of São Paulo is responsible for 54% of the country’s production [1,2].

The fleet growth in the motor pool, undesirable effects of fossil fuel (finite source), and other environmental concerns found ethanol and alcohol sector environmentally friendly fuel alternatives (biofuel) obtained from renewable sources (crops). Therefore, a prominent increase in the demand for ethanol has been observed around the world, especially in Brazil for having favorable edapho-climatic conditions for the large-scale cultivation and productive potential of sugarcane crops [3]. Also, sugarcane is a raw material to produce various other by-products for numerous economic sectors, such as food, cosmetics, and energy (by burning bagasse) which further contribute to environmental sustainability [4]. Most of the Brazilian sandy soils are newly occupied by sugarcane cultivation, which were often pasture degraded soil, generally limited with plant nutritional chemical attributes. Thus, adequate nutrient supply is necessary for well-nourished plants that reflect in higher yields with industrial quality and field longevity of sugarcane [5].

Phosphorus (P) is one of the most limiting nutrients in the tropical agricultural system, and at the same time, it is a major challenge to ensure P increased availability in the soil solution [6]. Phosphorus is a primary macronutrient with structural function in plants due to several metabolic processes, such as energy storage and transfer, respiration, nucleic acid synthesis, membrane tuning and stability, enzyme activation and deactivation, redox reactions, and carbohydrate metabolism [7]. It is essential for the synthesis of adenosine triphosphate (ATP) and several other phosphorylated compounds. The importance of P for plants lies in the fact that it participates directly and indirectly in various metabolic processes, thus acting from root development, tillering, stalk production, besides improving the industrial characteristics of sugarcane [8]. The low P availability is due to its high adsorption to the clay particles and its precipitation as Fe and Al [9] in clay fractions like amorphous hydrated oxides of Fe and Al, in addition to gibbsite, goethite and kaolinite are responsible for the greater P fixation for plant growth [7]. Approximately 70% of the phosphate fertilization in sugarcane is not available for plant use, requiring large amounts of fertilizers to achieve high yields [10].

Under tropical soil conditions, the P_2_O_5_ rate recommended for sugarcane production ranges from 130 to 180 kg ha^−1^ of P_2_O_5_ in planting [11,12,13]. Sugarcane can export approximately 30 kg P_2_O_5_ to produce 100 Mg ha^−1^ of stalks [14]. The high adsorption of P on Fe and Al oxides/hydroxides, which normally adsorb most of the P applied in the soil [15], explains the high rate of P applied at sugarcane fields in Brazil. Withers et al. [16] showed that in acid tropical soil, a rate of 80 kg ha^−1^ P_2_O_5_ is required to overcome soil P adsorption. The P fertilizers, commonly used, are soluble and require extensive amounts of phosphate rock and acids for their production. The conventional soluble P fertilizers should be substituted with renewable sources to build a circular economy, especially considering the recent evidence of excessive P use in tropical areas [16]. Therefore, search for new techniques aiming to increase phosphate fertilization efficiency, especially in regions of less fertility to reduce phosphate fertilization application and cost input, also reduce environmental impacts such as eutrophication of water sources [17]. Microbial inoculants, known as plant growth-promoting bacteria (PGPBs), are promising technologies to reduce the use of conventional synthetic fertilizers [18]. Thus, the association of sugarcane cultivation and PGPBs inoculation is an innovative approach to reduce phosphate fertilization while increasing the sugarcane yield with a positive impact on the circular economy in the Brazilian sugarcane industry, which represents more than one-third of all world production. In this sense, PGPBs could be one of the best alternatives for more sustainable agriculture, as they could promote fertilizer savings and other inputs and increase crop yield [12]. There are reports that these microorganisms can contribute in different ways to plant growth through the synthesis of phytohormones, secondary metabolites, root development, enhanced water and nutrients uptake, and solubilization of nutrients such as P [19] and zinc (Zn) [20]. Also, PGPBs are related to the promotion of biological nitrogen fixation (BNF), induction of tolerance to biotic and abiotic stresses, such as resistance to disease, pathogens attack, and other stresses [21,22,23,24,25,26].

*Azospirillum*, *Azobacter*, *Bacillus*, *Burkholderia*, *Gluconacetobacter*, *Herbaspirillum*, *Pseudomonas,* and *Rhizobium* are among the different genus of PGPBs found or associated with sugarcane crop [27,28]. Among these, microbial consortia containing the species, *Azospirillum brasilense*, and *Bacillus subtilis* have shown great potential to cycle nutrients from crop residues and restore soil fertility, and improve the sugarcane rhizosphere [12,27]. In addition, *Bacillus* and *Pseudomonas* genera are among the most efficient solubilizers of inorganic phosphate [29], which could contribute to reducing phosphate application under agricultural systems. Over the last decade, the inoculants market has grown substantially worldwide. In Brazil, an impressive increase in the number of commercialized PGPBs has been seen in the short time of a decade, with commercial inoculants with *A. brasilense*, *B. subtilis*, and *P. fluorescens* being released. Understanding the success or failure of inoculation requires understanding the complex interactions between plant species, the specificity between hosts and PGPB, and the major microbial communities in the rhizosphere [30,31]. Therefore, studies with different PGPB inoculation such as *A. brasilense*, *B. subtilis,* and *P. fluorescens* in tropical conditions should be performed since new reports can be largely applicable to other important producing countries. We hypothesized that phosphate fertilization in field-grown sugarcane plants could be improved by *A. brasilense*, *B. subtilis,* and *P. fluorescens* inoculations coupled with P_2_O_5_ application rates, providing increased sugarcane yield and industrial quality with reduced P_2_O_5_ rates. Thus, the results would provide a novel perspective for sustainable sugarcane cultivation under tropical conditions by improving P fertilizer use and uptake without compromising sugarcane yield and quality. The objective of this study was to evaluate the effect of *A. brasilense*, *B. subtilis*, and *P. fluorescens* inoculation in a single or combined application associated with P_2_O_5_ application rates, on nitrogen (N) and P leaf concentrations, stalk and sugar yield, and technological quality of sugarcane under tropical conditions.

## 2. Materials and Methods

### 2.1. Experimental Area Location

**Figure 1:**
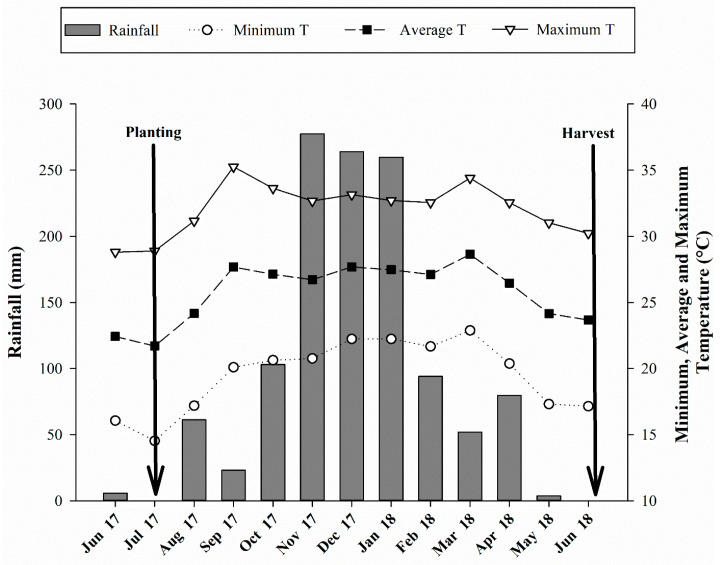
Monthly average rainfall and maximum, average, and minimum temperature recorded during the period of experiment.

The experiment was carried out in the 2017/2018 crop season at Limoeiro Farm in an area belonging to Suzanápolis Sugar and Alcohol Mill in Ilha Solteira, located in the northwestern State of São Paulo, Brazil (Appendix A). The geographical coordinates are 20°21′14″ S latitude and 51°04′51″ W longitude with 371 m above sea level. The experimental area has been cultivated with highly degraded pasture (*Urochloa brizantha*) for over 10 years. The soil of the agricultural area was classified as a Latossolo Vermelho distrófico, from medium to sandy texture according to SiBCS [32], and Rhodic Haplustox according to the Soil Survey Staff [33]. The climate of the region is Aw, according to the Köppen scale, defined as humid tropical with a rainy season in summer and dry in winter. The climatic data regarding the experiment conduction period were properly recorded and are shown in Figure 1. The granulometry analysis showed 777, 98, 125 g kg^−1^ and 747, 88, 165 g kg^−1^ of sand, silt, and clay at the layers of 0.00–0.25 and 0.25–0.50 m, respectively, following the methodology of Embrapa [34]. The chemical attributes of the soil were determined before the experiment implementation and are shown in Table 1.

### 2.2. Experimental Design and Treatments

The experimental design was a randomized block design with three replications, arranged in an 8 × 5 factorial scheme. Eight inoculations ((1) Control (without inoculation); (2) Inoculation with *Azospirillum brasilense*; (3) Inoculation with *Bacillus subtilis*; (4) Inoculation with *Pseudomonas fluorescens*; (5) Inoculation with *A. brasilense* + *B. subtilis*; (6) Inoculation with *A*. *brasilense* + *P. fluorescens*; (7) Inoculation with *B. subtilis* + *P. fluorescens*; (8) Inoculation with *A. brasilense* + *B. subtilis* + *P. fluorescens*) and five P_2_O_5_ rates (0, 45, 90, 135, and 180 kg ha^−1^) hand applied in the planting furrows. These PGPBs are commercial strains used in Brazil [for both *A. brasilense* (brand name AzoTotal^®^), *B. subtilis* (brand name Vult^®^), and *P. fluorescens* (brand name Audax^®^)]. These strains when used under similar conditions (Brazilian tropical conditions) have shown positive results in maize and soybean [35,36]. Triple superphosphate (46% of P_2_O_5_) was used as a P_2_O_5_ source, corresponding to 0, 25, 50, 75, and 100% of the recommended rate, according to van Raij [11]. The plots had five 5.0 m long rows with spacing of 1.5 m, and three central rows were considered as useful plot areas.

### 2.3. Experiment Implementation and Conduction

After initial soil chemical characterization, the soil profile was prepared by applying 1.0 t ha^−1^ of dolomitic limestone with engineered cementitious composites (ECC) of 85% to increase base saturation to 60% 15 days before planting. Also, 1.0 t ha^−1^ of gypsum (15% of S) was applied to increase sulfur content (S) following the recommendation of van Raij [11] for sugarcane cultivation. Three plowing and one subsoiling operation were performed, followed by the opening of planting furrow at 0.40 m depth, and insecticide—fipronil (180 g of active ingredient [a.i.] ha^−1^) + fungicide—pyraclostrobin (125 g a.i. ha^−1^) application.

The stems of the RB92579 variety were manually cultivated on 11 July 2017, and sectioned within the planting furrows containing about 22 buds per meter of the furrow. In the planting fertilization, besides the respective rates of P_2_O_5_ of each treatment, 30 kg ha^−1^ of N (as ammonium nitrate—34% of N) and 120 kg ha^−1^ of K_2_O (as potassium chloride—60% of K_2_O) were equally applied to all treatments, based on soil analysis.

The inoculations were performed by spraying PGPBs with a manual sprayer pump at a volume of 200 L ha^−1^ in the late afternoon due to the milder temperature in the planting furrow. The following rates of liquid inoculant were sprayed: 1.0 L ha^−1^ for *A. brasilense* (strains Ab-V5 and Ab-V6—the guarantee of 2 × 10^8^ colony forming units (CFU) mL^−1^); 0.5 L ha^−1^ for *B. subtilis* (strain CCTB04 guarantee of 1 × 10^8^ CFU mL^−1^) and 0.5 L ha^−1^ for *P. fluorescens* (strain CCTB03 guarantee of 1 × 10^8^ CFU mL^−1^) based on the manufacturer’s recommendation. The furrows were mechanically covered after the application of the bacteria (treatments). Due to the absence of rainfall during the experiment implementation, the experimental area was irrigated with sprinkler irrigation (water depth of 30 mm) in the very first five days after sugarcane planting.

In the sugarcane tillering phase (at 123 DAP—days after planting) all plots were fertilized with 5.0 kg Zn ha^−1^ (as zinc sulfate—21% of Zn and 11% of S), applied on the soil surface to fulfill zinc-deficient content in the soil.

Control of weeds, pests, and diseases was performed according to crop needs. Hexazinone (320 g a.i. ha^−1^) + 2.4 D (967 g a.i. ha^−1^) + tebuthiuron (1000 g a.i. ha^−1^) were applied in pre-emergence (24 DAP). At 110 DAP, 2.4 D (967 g a.i. ha^−1^) + amicarbazone (840 g a.i. ha^−1^) + S-Metolachlor (2400 g a.i. ha^−1^) were applied. At 101 and 199 DAP, insecticides chlorantraniliprole (10 g a.i. ha^−1^) + lambda-cyhalothrin (5 g a.i. ha^−1^) were applied. Aiming at the biological control of the sugarcane borer (*Diatraea saccharalis*), four releases of *Trichogramma galloi* (142, 148, 213, and 220 DAP, respectively) and one release of *Cotesia flavipes* (192 DAP) were performed. At 301 DAP, the Trinexapaque-ethyl ripener (275 g a.i. ha^−1^) was applied to induce sugarcane to accumulate sucrose. The plant-cane harvest was carried out manually on 25 June 2018, 349 days after planting.

### 2.4. Evaluations

In the phase of more intense vegetative development of sugarcane (195 DAP), the average third of 15 flag leaves per plot were collected, excluding the midrib by following the methodology of van Raij [11] to determine N and P leaf concentrations as described by Malavolta [37]. Briefly, N was analyzed by sulfuric digestion, distilled by semi-micro Kjeldahl method, and determined by titration with HCl 0.05 N. Phosphorus was analyzed by nitro-perchloric digestion and quantified in an Ultraviolet-visible spectroscopy spectrophotometer (UV-VIS—Model Varian Cary-50, manufacturer Varian, city Victoria, country Australia).

At the harvest (349 DAP), the stem mass of each plot was quantified to estimate stalk yield per hectare (STY—t ha^−1^). Also, at the time of harvest, ten stalks were sampled per plot and sent to the Technological Analysis Laboratory to determine the technological quality of sugarcane according to the methodology defined in the System Sugarcane Payment Plan based on Sucrose Content (SSPPSC) following Fernandes [38] methodology. Fiber (%), purity apparent of the juice (%), soluble solids content (Brix—%), apparent sucrose of juice (Pol—%), and total recoverable sugar (TRS—kg t^−1^; TRS is characterized as the sum of sucrose that can be converted into sugar or alcohol) were determined at Suzanápolis Sugar and Alcohol Mill laboratory. The sequence of technological quality analysis also can be assessed in Lopes et al. [13]. The sugar yield (SUY—t ha^−1^) was obtained by the multiplication of stalk yield per hectare and sugarcane pol % of each plot, divided by 100.

### 2.5. Statistical Analysis

The data were submitted to analysis of variance (F test) and Scott Knott test (*p* ≤ 0.05) to group the inoculation means and adjust the regression equations for the effect of P_2_O_5_ rates. Statistical analyses were processed using the R software [39], and graphs were plotted with the aid of SigmaPlot 14.5 software (Systat Software, San Jose, CA, USA).

## 3. Results

### 3.1. Nitrogen and P Leaf Concentrations

Bacterial inoculation and P_2_O_5_ rates did not influence N leaf concentration in sugarcane crops (Table 2). However, single inoculation with *P. fluorescens* and co-inoculation except *A. brasilense* + *P. fluorescens* provided higher leaf P concentration regardless of P_2_O_5_ rates applied (Table 2). These inoculations (e.g., *P. fluorescens*, *A. brasilense* + *B. subtilis*, and *A. brasilense* + *B. subtilis* + *P. fluorescens*) increased leaf P concentration, varying between 4.4% and 10.2% compared to control treatments (Table 2).

### 3.2. Sugarcane Technological Quality

**Figure 2:**
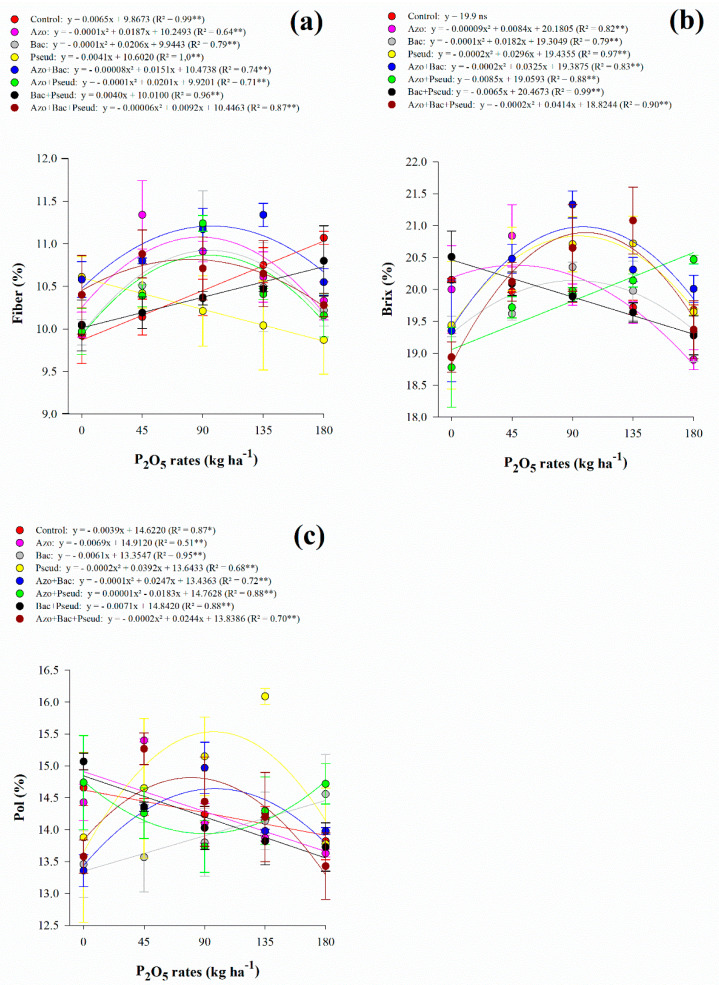
Interaction between P_2_O_5_ rates and inoculations for fiber (**a**), soluble solids content (Brix) (**b**) and apparent sucrose of juice (Pol) (**c**) content of sugarcane. Error bars indicate the standard deviation of the mean (*n* = 3). **, * and ns: significant at 1% at *p* < 0.01, 5% at *p* < 0.05 and *p* > 0.01 and not significant, respectively.

Interaction between P_2_O_5_ rates and inoculations was significant for fiber content, Brix, Pol, and TRS (Table 3). However, the apparent purity of juice was not influenced by any of the variable sources and their interactions (Table 3). Regarding fiber content, there was a non-linear adjustment for *A. brasilense* (up to 94 kg P_2_O_5_ ha^−1^), *B. subtilis* (up to 103 kg P_2_O_5_ ha^−1^), *A. brasilense* + *B. subtilis* (up to 94 kg P_2_O_5_ ha^−1^), *A. brasilense* + *P. fluorescens* (up to 101 kg P_2_O_5_ ha^−1^) and *A. brasilense* + *B. subtilis* + *P. fluorescens* (up to 77 kg P_2_O_5_ ha^−1^) inoculations (Figure 2a). Inoculation with *B. subtilis* + *P. fluorescens* and control treatment indicated linear adjustment with increasing P_2_O_5_ rates whereas *P. fluorescens* decreased with increasing P_2_O_5_ rates (Figure 2a). Fiber content fluctuated with all applied P_2_O_5_ rates and inoculations however, the lowest content was associated with high P_2_O_5_ rates (180 kg ha^−1^) along single inoculation of *A. brasilense*, *B. subtilis*, and *P. fluorescens* and co-inoculation of *A. brasilense* + *P. fluorescens* and *A. brasilense* + *B. subtilis* + *P. fluorescens* (Table 4).

**Figure 3:**
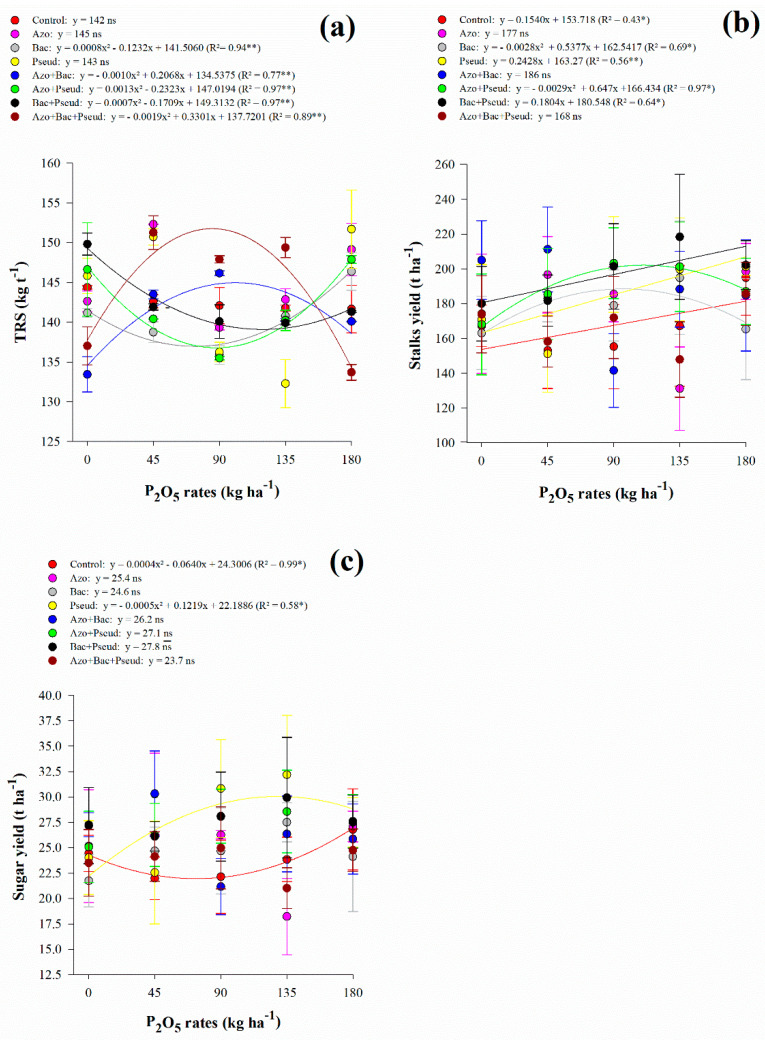
Interaction between P_2_O_5_ rates and inoculations in total recoverable sugars (TRS) (**a**), stalks yield (STY) (**b**) and sugar yield (SUY) (**c**). Error bars indicate the standard deviation of the mean (*n* = 3). **, * and ns: significant at 1% at *p* < 0.01, 5% at *p* < 0.05 and *p* > 0.01 and not significant, respectively.

The soluble solids content of juice responded non-linearly to increasing P_2_O_5_ rates in *A. brasilense* (up to 47 kg P_2_O_5_ ha^−1^), *B. subtilis* (up to 91 kg P_2_O_5_ ha^−1^), *P. fluorescens* (up to 74 kg P_2_O_5_ ha^−1^), *A. brasilense* + *B. subtilis* (up to 81 kg P_2_O_5_ ha^−1^) and *A. brasilense* + *B. subtilis* + *P. fluorescens* (up to 104 kg P_2_O_5_ ha^−1^) inoculations (Figure 2b). Inoculation with *A. brasilense* + *P. fluorescens* showed linear adjustment with increasing P_2_O_5_ rates while *B. subtilis* + *P. fluorescens* decreased with increasing P_2_O_5_ rates (Figure 2b). Apparent sucrose of juice responded non-linearly to increasing P_2_O_5_ rates in *P. fluorescens* (up to 98 kg P_2_O_5_ ha^−1^), *A. brasilense* + *B. subtilis* (up to 124 kg P_2_O_5_ ha^−1^), *A. brasilense* + *P. fluorescens* (Pmin = 91.5 kg P_2_O_5_ ha^−1^), and *A. brasilense* + *B. subtilis* + *P. fluorescens* (up to 61 kg P_2_O_5_ ha^−1^) inoculations (Figure 2c). Inoculation with *B. subtilis* showed linear adjustment with increasing P_2_O_5_ rates while *A. brasilense*, *B. subtilis* + *P. fluorescens*, and control treatment decreased with increasing P_2_O_5_ rates (Figure 2c). Total recoverable sugar responded non-linearly to increasing P_2_O_5_ rates in B. subtilis (Pmin = 77 kg P_2_O_5_ ha^−1^), *A. brasilense* + *P. fluorescens* (Pmin = 89 kg P_2_O_5_ ha^−1^), *B. subtilis* + *P. fluorescens* (Pmin = 122 kg P_2_O_5_ ha^−1^), *A. brasilense* + *B. subtilis* (up to 103 kg P_2_O_5_ ha^−1^) and *A. brasilense* + *B. subtilis* + *P. fluorescens* (up to 87 kg P_2_O_5_ ha^−1^) inoculations) (Figure 3a). In general, under low (45 kg ha^−1^), average (90 and 135 kg ha^−1^), and high (180 kg ha^−1^) P_2_O_5_ rates associated with single and combined inoculations with *A. brasilense*, *B. subtilis*, and *P. fluorescens* provided greater Brix, Pol, and TRS when compared to control treatment (Table 4).

### 3.3. Stalk and Sugar Yield

Interaction between P_2_O_5_ rates and inoculations was significant for STY and SUY (Table 2). There was a linear adjustment with increasing P_2_O_5_ rates for control treatment, *P. fluorescens* and *B. subtilis* + *P. fluorescens* inoculation on STY, whereas a non-linear adjustment was verified for *B. subtilis* (up to 96 kg P_2_O_5_ ha^−1^), and *A. brasilense* + *P. fluorescens* (up to 112 kg P_2_O_5_ ha^−1^) (Figure 3b). In general, we did not verify a specific trend of inoculations however low (45 kg ha^−1^) and average (90 and 135 kg ha^−1^) P_2_O_5_ rates along with single and/or combined inoculations of *A. brasilense*, *B. subtilis*, and *P. fluorescens* except the combination of all three bacteria provided greater STY compared to control treatment (Figure 3b). The highest STY was verified with *A. brasilense* + *B. subtilis* inoculation associated with 45 kg P_2_O_5_ ha ha^−1^ providing 211 t STY ha ha^−1^, reflecting an increase of 38% compared to control treatment (Table 5). Also, inoculation with *A. brasilense* + *B. subtilis* associated with 45 kg P_2_O_5_ ha ha^−1^ provided an increase of 11.1% when compared to the average of all inoculations and control treatments associated with 180 kg P_2_O_5_ ha ha^−1^ (Table 5).

Similarly, SUY was greater under low (45 kg ha^−1^) and average (90 and 135 kg ha^−1^) P_2_O_5_ rates associated with single and combined inoculations of *A. brasilense*, *B. subtilis*, and *P. fluorescens* except for the combination of all three bacteria when compared to control treatment (Figure 3c). The highest SUY (32.19 t ha^−1^) was verified with *P. fluorescens* inoculation under 135 kg P_2_O_5_ ha^−1^ followed by the inoculation of *A. brasilense* + *B. subtilis* associated with 45 kg P_2_O_5_ ha^−1^ (30.33 t ha^−1^) (Figure 3c). These treatments provided an increase of 21.9% (*P. fluorescens* associated with 135 kg P_2_O_5_ ha^−1^) and 14.9% (*A. brasilense* + *B. subtilis* associated with 45 kg P_2_O_5_ ha^−1^) when compared to the average of all inoculations and control treatment associated with 180 kg P_2_O_5_ ha^−1^ (Table 5). In complement, SUY showed a non-linear adjustment for control treatment (Pmin = 80 kg P_2_O_5_ ha^−1^) and *P. fluorescens* inoculation (up to 122 kg P_2_O_5_ ha^−1^) (Table 5).

## 4. Discussion

Our results indicated that PGPB inoculation favored P absorption as verified by increased leaf P concentration with *P. fluorescens* inoculation (an increase of 7.1%), *A. brasilense* + *B. subtilis* (an increase of 5.3%), *B. subtilis* + *P. fluorescens* (an increase of 10.2%) and *A. brasilense* + *B. subtilis* + *P. fluorescens* (an increase of 4.4%), respectively. Although leaf N concentration was not greatly influenced by inoculations, the same above-mentioned treatments provided a numerical increase on N concentration varying between 2.6% to 5.7%. The N concentration (varying between 19.5 and 21.1 g N kg^−1^) and P concentration in sugarcane leaf tissue (varying between 2.2 and 2.5 g P kg^−1^) were in the suitable concentration range for N (18–25 g kg^−1^ D.M.) and P (1.5–3.0 g kg^−1^ of D.M.) according to van Raij [11]. Phosphorus serves as an energy booster for the activation of certain endergonic activities including, nutrients assimilation, organic compounds production [40,41], and also root-shoot growth of the plants [42,43]. Phosphorus has several benefits in tropical sugarcane crops [44,45], decreasing unavailable soil P content by increasing its availability to sugarcane plants under low P fertilizer rates [16]. Several PGPB can increase plant P availability through phosphate solubilization by adapting mechanisms such as acidification, chelation, organic acid production, secreting acid phosphatase that mineralizes organic-P [46]. In addition, some PGPBs can mineralize organic sources of P through the action of phytases, as well as acid and alkaline phosphatases [47,48]. They can also prevent P adsorption under conditions limiting P nutrient by assimilating solubilized phosphate. Thus, these microorganisms can act as a reservoir and supply P to plants upon their demand [46]. Singh et al. [49] exhibited that effective N-fixing bacteria more prominently increased certain physiological and growth activities of sugarcane, P accessibility, synthesis of indole acetic acid producers. While Beneduzi et al. [50], demonstrated that phosphate solubilizing bacteria and indole acetic acid producers in sugarcane promoted a beneficial effect on plant-bacteria interaction. Therefore, the inoculation of plants with more than one species of plant growth-promoting bacteria, combined with mineral fertilization is interesting and results in mechanisms that increase sugarcane nutrition, yield, and quality. This is anticipated to happen due to the impact of different quantitative modes of action and may ensure that at most one mode of action is explicated in a specific agro-system [51], and indeed, multi-functional strains are typically the most efficient PGPB [52].

Previous studies performed under controlled conditions already demonstrated the potential of PGPBs to improve sugarcane yields [53]. Also, the positive effect of PGPBs on plant biomass and nutrition has been observed in soils with low P availability [48]. Magallon-Servin et al. [54] verified an increase in N, P, and K uptake by maize plants due to the inoculation with PGPBs related to phosphate solubilization (PSBs) in a low-P soil fertilized with rock phosphate. Similarly, the co-inoculation of bean plants with *Rhizobium* sp. and PSBs strains increased nodule weight, shoot dry weight, and N fixation when compared with the single inoculation with *Rhizobium* sp. [55]. In sugarcane field experiments, the co-inoculation of *A. brasilense* and *B. subtilis* also contributed to reducing P fertilization by 75% [12]. Lopes et al. [13] concluded that the application of sugarcane by-products enriched with phosphate rocks and PSBs (*P. aeruginosa* PSBR12, *Bacillus* sp. BACBR01, BACBR04, BACBR06, and *Rhizobium* sp. RIZBR01) is a great alternative to increase β-glucosidase activity and the content of labile and moderately labile P fractions in tropical soils. Estrada-Bonilla et al. [48] verified that the use of compost (filter cake and ashes) and PSBs (*Bacillus* sp. BACBR04, *Bacillus* sp. BACBR06, and *Rhizobium* sp. RIZBR01) improved soil P availability and positively affected sugarcane plant nutrition mainly due to an increase in the abundance of phytate-degrading enzymes correlated with the improvement of organic labile P in soil when PSBs are co-inoculated. This evidence further supports the potential of PGPBs to increase sugarcane plant nutrition by enhancing P-fertilization even with low solubility P sources and improving soil P lability. Nonetheless, the inoculation with PGPBs probably improved the physiological attributes of the plant since inoculation with microorganisms has the potential to increase plant and root growth [18]. Increased root biomass can positively influence root scavenging, which is important for the interception of nutrients in crop systems such as P leading to a greater P-fertilizer use efficiency (PUE). For example, Pereira et al. [36] concluded that inoculation with *A. brasilense* and *B. subtilis* can increase P uptake, benefiting productive components development, leading to an improved PUE, and greater maize grain yield. According to these authors, P uptake and PUE increased 100.5 and 54.6% as a result of *A. brasilense* and *B. subtilis* inoculation compared to non-inoculated treatments. Therefore, further investigation related to PGPBs roles, and the key mechanisms related to phosphate solubilization and PUE should be performed.

Although PGPB inoculation differently responds under P_2_O_5_ application rates, our results showed that combinations of PGPBs inoculation provided greater sugarcane technological quality. We verified that response to single inoculation of *P. fluorescens* and *A. brasilense* + *B. subtilis* associated with low (45 kg P_2_O_5_ ha^−1^) and average (90 and 135 kg P_2_O_5_ ha^−1^) rates provided greater Brix and Pol. Also, Brix and Pol content were greater with *A. brasilense* + *B. subtilis* when associated with a high P_2_O_5_ rate (180 kg ha^−1^). Fiber content was not greatly influenced by inoculations and P_2_O_5_ rates. However, when 180 kg P_2_O_5_ ha^−1^ was applied together with single inoculation of *A. brasilense*, *P. fluorescens*, and *B. subtilis* and combinations of *A. brasilense* and *P. fluorescens* and *A. brasilense* + *B. subtilis* and *P. fluorescens* reduced fiber content between 2.4% and 10.8%. The improved Brix and Pol content, together with reduced fiber content, led to a greater TRS. In the absence of P_2_O_5_ fertilization, *B. subtilis* + *P. fluorescens* provided 3.8% greater TRS compared to control (non-inoculated) treatments. The low and average P_2_O_5_ application rates (45, 90, and 135 kg P_2_O_5_ ha^−1^) together with *A. brasilense* + *B. subtilis* + *P. fluorescens* inoculation increased TRS by 6.1%, 4.1%, and 5.4%, respectively, compared to control treatments. The application of higher P_2_O_5_ rates (180 kg ha^−1^) together with *P. fluorescens* inoculation provided an increase of 7.1% in TRS, compared to control treatment.

Sugarcane yield was also benefited with PGPB inoculation and P_2_O_5_ rates. Stalk and sugar yield showed greater production with inoculation of *A. brasilense* and *A. brasilense* + *B. subtilis* when associated with 45 kg P_2_O_5_ ha^−1^. The application of 90 kg P_2_O_5_ ha^−1^ along with single inoculation of *A. brasilense* and *P. fluorescens* and combined inoculation of *A. brasilense* + *P. fluorescens* and *B. subtilis* + *P. fluorescens* provided greater STY and SUY. Similarly, application of 135 kg P_2_O_5_ ha^−1^ in association with single inoculation of *B. subtilis* and *P. fluorescens* and combined inoculation of *A. brasilense* + *B. subtilis*, *A. brasilense* + *P. fluorescens*, and *B. subtilis* + *P. fluorescens* provided greater STY and SUY. Our results showed that the application of P_2_O_5_ at the rate of 180 kg ha^−1^ without inoculation provided greater STY and SUY. However, inoculations with *A. brasilense* + *B. subtilis* increased stalk yield with a reduction of 75% in P_2_O_5_ application rates (180 to 45 kg P_2_O_5_ ha^−1^). We verified an increase of 8.4% in stalk yield with inoculation of *A. brasilense* + *B. subtilis* under the treatment of 45 kg P_2_O_5_ ha^−1^ when compared to the application of 180 kg P_2_O_5_ ha^−1^ without inoculation. In addition, *A. brasilense* + *B. subtilis* inoculation and 45 kg P_2_O_5_ ha^−1^ provided an increase of 13.2% in sugarcane yield compared to the application of 180 kg P_2_O_5_ ha^−1^ without inoculation.

The exact mechanisms underlying the effect of *A. brasilense* and *B. subtilis* on sugarcane yield and quality were not evaluated in the present study. However, the current field trail verified that increased P availability and uptake along with the inoculation of *A. brasilense* and *B. subtilis* were prominently observed in the form of high leaf P content, growth, industrial quality, and yield of sugarcane crops [56,57,58,59,60]. The gene sequence of *A. brasilense* (strains Ab-V5 and Ab-V6) highlighted its role in auxins synthesis [29], increased availability of nutrients [61], and BNF [62,63]. In addition, *B. subtilis* is described with the potential ability to promote plant growth, P solubilization, and inhibit infestation of phytopathogenic attack and heavy metal accumulation [64,65,66]. *Bacillus* sp. is a genus that is characterized for being an excellent phosphate solubilizer, including the species of *B. subtilis* which becomes essential under the consideration that P is one of the most critical nutrients to increase crop yields [67,68]. The studies exhibited that under the inoculation of *A. brasilense* and *B. subtilis*, plants may adapt different mechanisms to more potentially scrutinize and uptake soil P [58,66,68].

The single inoculation with *P. fluorescens* and/or in combination with *A. brasilense* and *B. subtilis* also benefited leaf P concentration, industrial quality, and yield of sugarcane. *Pseudomonas fluorescens* is presently considered the most efficient bacteria, using secondary metabolites as a bio-control agent with the synthesis of antibiotics, volatile organic compounds to combat soil pathogens [69,70], promising phosphate solubilization [71] and N fixation activities [72]. According to Pineda [73], luconic acid is among the different types of organic acids involved in phosphate solubilization which is produced by *Pseudomonas* sp. Thus, new studies assessing multiple effects of different PGPB applied in combination should be performed to improve P_2_O_5_-based fertilizers application aiming at improved sustainable sugarcane production systems.

## 5. Conclusions

Inoculation with *Bacillus subtilis* + *Pseudomonas fluorescens* provided the highest leaf concentration of P in the diagnostic leaf of plant-cane, RB92579 variety.

The combination of *Azospirillum brasilense* with the application of 45 kg P_2_O_5_ ha^−1^ promoted the best agro-industrial quality of sugarcane.

In the absence of phosphate fertilizer and the highest rate of P_2_O_5_ (180 kg ha^−1^), stalk yield (STY) and sugar yield (SUY) were not positively influenced by inoculation with PGPBs, alone or in combination, demonstrating that extremes were detrimental to obtain satisfactory results.

Inoculation with *Azospirillum brasilense* + *Bacillus subtilis* associated with 45 kg P_2_O_5_ ha^−1^ at planting is recommended, as this treatment increases the yield of sugarcane stalks by 38% when compared to the control treatment (without inoculation), at the same rate, in soil with low P content. This treatment also increases sugar yield, resulting in a savings of 75% of the recommended P_2_O_5_ rate, thus being a more efficient and sustainable alternative for reducing sugarcane crop production costs.

## Figures and Tables

**Table 1:** Initial soil chemical characterization ^a^ of the experimental area in the 0.00–0.25 and 0.25–0.50 m layers.

Layer	P Resin	S-SO_4_	OM		pH		K	Ca		Mg		H + Al		Al	SB
(m)	-----mg dm^−3^-----		g dm^−3^		CaCl_2_		--------------------------mmol ^c^ dm^−3^------------------------								
0.00–0.25	2	3	13		4.7		2.6	8		6		20		1	16.6
0.25–0.50	2	2	12		4.8		2.4	9		7		20		2	18.4
Layer	B ^b^	Cu ^c^		Fe ^c^		Mn ^c^			Zn ^c^		CEC		V		m
(m)	--------------------------mg dm^−3^--------------------------										mmol ^c^ dm^−3^		%		%
0.00–0.25	0.22	0.8		14		16.2			0.6		36.6		45		6
0.25–0.50	0.22	1.0		7		8.3			0.3		38.4		48		10

^a^ Methodology proposed by van Raij et al. [11], ^b^ Determined in DTPA (diethylenetriaminepentaacetic acid), ^c^ Determined in hot water. OM: organic matter, CEC: cation exchange capacity, SB: sum of bases, V: bases saturation, m: Al saturation.

**Table 2:** Nitrogen and phosphorus concentrations in sugarcane diagnose leaf.

	Nitrogen	Phosphorus
	g kg^−^^1^	
Rates of P_2_O_5_		
(kg ha^−1^)		
0	20.44	2.32
45	20.53	2.34
90	20.08	2.37
135	20.39	2.31
180	20.33	2.36
Inoculation		
Control	19.53	2.25 b
Azo	20.09	2.22 b
Bac	21.14	2.32 b
Pseud	20.58	2.41 a
Azo + Bac	20.03	2.37 a
Azo + Pseud	20.36	2.31 b
Bac + Pseud	20.65	2.48 a
Azo + Bac + Pseud	20.47	2.35 a
F test		
Rates of P_2_O_5_ (D)	ns	ns
Inoculation (I)	ns	*
D × I	ns	ns
Overall Average	20.36	2.34
Standard error	0.34	0.05

Averages followed by the same letter in the columns belong to the same group by Scott and Knott test at 5% probability. Control (without inoculation); Azo (*Azospirillum brasilense*); Bac (*Bacillus subtilis*); Pseud (*Pseudomonas fluorescens*); Azo + Bac (*A. brasilense* + *B. subtilis*); Azo + Pseud (*A. brasilense* + *P. fluorescens*); Bac + Pseud (*B. subtilis* + *P. fluorescens*); Azo + Bac + Pseud (*A. brasilense* + *B. subtilis* + *P. fluorescens*). CV: coefficient of variation. * and ns: significant at 5% at *p* < 0.05 and *p* > 0.01 and not significant, respectively.

**Table 3:** Indicators of technological quality, stalk yield (STY) and sugar yield (SUY) of RB92579 sugarcane variety.

	Fiber	Purity	Brix	Pol	TRS	STY	SUY
	%	%	%	%	kg t^−1^	t ha^−1^	t ha^−1^
Rates of P_2_O_5_							
(kg ha^−1^)							
0	10.19	81.58	19.57	14.15	142.62	175.31	24.80
45	10.58	82.12	20.16	14.53	145.20	177.60	25.80
90	10.77	81.28	20.34	14.31	140.41	180.11	25.78
135	10.59	81.03	20.15	14.33	140.95	181.14	25.96
180	10.41	82.84	19.59	13.96	143.99	189.99	26.41
Inoculation							
Control	10.45	82.27	19.89	14.27	142.51	167.58	23.85
Azo	10.65	83.72	19.86	14.29	145.26	177.23	25.41
Bac	10.48	81.21	19.74	13.91	140.60	177.11	24.55
Pseud	10.23	82.05	20.20	14.71	143.37	185.13	27.42
Azo + Bac	10.89	81.60	20.29	14.13	140.70	186.21	26.19
Azo + Pseud	10.43	81.85	19.82	14.35	142.14	189.05	27.13
Bac + Pseud	10.37	80.74	19.88	14.20	142.62	196.78	27.77
Azo + Bac + Pseud	10.58	80.72	20.03	14.18	143.87	167.56	23.68
F test							
Rates of P_2_O_5_ (D)	**	ns	**	**	**	ns	ns
Inoculation (I)	**	ns	**	**	**	**	**
D × I	**	ns	**	**	**	**	**
Overall Average	10.51	81.77	19.96	14.25	142.63	180.83	25.75
Standard error	0.07	0.86	0.09	0.12	0.49	5.30	0.75
CV (%)	2.59	4.09	1.87	3.23	1.33	13.11	13.10

TRS: Total recoverable sugar. Averages followed by the same letter in the columns belong to the same group by the Scott and Knott test, at 5% probability. Control (without inoculation); Azo (*Azospirillum brasilense*); Bac (*Bacillus subtilis*); Pseud (*Pseudomonas fluorescens*); Azo + Bac (*A. brasilense* + *B. subtilis*); Azo + Pseud (*A. brasilense* + *P. fluorescens*); Bac + Pseud (*B. subtilis* + *P. fluorescens*); Azo + Bac + Pseud (*A. brasilense* + *B. subtilis* + *P. fluorescens*). CV: coefficient of variation. ** and ns: significant at 1% at *p* < 0.01 and not significant, respectively.

**Table 4:** Interaction of inoculation and P_2_O_5_ rates for fiber, Brix and pol in RB92579 sugarcane variety.

	**Fiber (%)**				
	**Rates of P_2_O_5_ (kg ha^−1^)**				
**Inoculation**	**0**	**45**	**90**	**135**	**180**
Control	9.92 b	10.14 c	10.37 b	10.75 b	11.07 a
Azo	10.05 b	11.34 a	10.91 a	10.61 b	10.33 b
Bac	9.96 b	10.51 c	11.22 a	10.49 b	10.21 b
Pseud	10.61 a	10.42 c	10.21 b	10.04 b	9.87 b
Azo + Bac	10.58 a	10.80 b	11.17 a	11.34 a	10.55 a
Azo + Pseud	9.97 b	10.39 c	11.24 a	10.41 b	10.16 b
Bac + Pseud	10.04 b	10.19 c	10.36 b	10.47 b	10.80 a
Azo + Bac + Pseud	10.40 a	10.88 b	10.71 b	10.65 b	10.28 b
Standard error	0.16				
	**Brix (%)**				
	**Rates of P_2_O_5_ (kg ha^−1^)**				
**Inoculation**	**0**	**45**	**90**	**135**	**180**
Control	20.15 a	19.97 b	19.90 c	19.72 b	19.68 b
Azo	20.00 a	20.84 a	19.92 c	19.64 b	18.90 b
Bac	19.42 b	19.62 b	20.35 c	19.98 b	19.33 b
Pseud	19.44 b	20.48 a	20.71 b	20.72 a	19.65 b
Azo + Bac	19.35 b	20.48 a	21.33 a	20.31 b	20.01 a
Azo + Pseud	18.78 c	19.72 b	19.98 c	20.14 b	20.47 a
Bac + Pseud	20.51 a	20.09 b	19.89 c	19.64 b	19.28 b
Azo + Bac + Pseud	18.94 c	20.12 b	20.65 b	21.08 a	19.37 b
Standard error			0.22		
	**Pol (%)**				
	**Rates of P_2_O_5_ (kg ha^−1^)**				
**Inoculation**	**0**	**45**	**90**	**135**	**180**
Control	14.66 a	14.36 b	14.24 b	14.26 b	13.82 b
Azo	14.43 a	15.40 a	14.10 b	13.87 b	13.63 b
Bac	13.46 b	13.57 b	13.81 b	14.14 b	14.56 a
Pseud	13.88 b	14.65 b	15.15 a	16.10 a	13.78 b
Azo + Bac	13.36 b	14.34 b	14.97 a	13.98 b	13.98 b
Azo + Pseud	14.74 a	14.26 b	13.74 b	14.30 b	14.72 a
Bac + Pseud	15.07 a	14.36 b	14.03 b	13.82 b	13.73 b
Azo + Bac + Pseud	13.58 b	15.27 a	14.44 b	14.20 b	13.43 b
Standard error	0.27				

Averages followed by the same letter in the columns belong to the same group by the Scott and Knott test, at 5% probability. Control (without inoculation); Azo (*Azospirillum brasilense*); Bac (*Bacillus subtilis*); Pseud (*Pseudomonas fluorescens*); Azo + Bac (*A. brasilense* + *B. subtilis*); Azo + Pseud (*A. brasilense* + *P. fluorescens*); Bac + Pseud (*B. subtilis* + *P. fluorescens*); Azo + Bac + Pseud (*A. brasilense* + *B. subtilis* + *P. fluorescens*).

**Table 5:** Interaction of inoculation and P_2_O_5_ rates for total recoverable sugar (TRS), stalk yield (STY) and sugar yield (SUY) of RB92579 sugarcane variety.

	**TRS (kg t^−1^)**				
	**Rates of P_2_O_5_ (kg ha^−1^)**				
**Inoculation**	**0**	**45**	**90**	**135**	**180**
Control	144.38 c	142.63 b	142.10 b	141.78 b	141.68 c
Azo	142.64 c	152.33 a	139.33 c	142.86 b	149.15 b
Bac	141.18 c	138.75 c	135.90 d	140.81 b	146.39 b
Pseud	145.84 b	150.74 a	136.27 d	132.29 c	151.71 a
Azo + Bac	133.45 e	143.51 b	146.18 a	140.28 b	140.10 c
Azo + Pseud	146.64 b	140.43 c	135.50 d	140.24 b	147.89 b
Bac + Pseud	149.83 a	141.92 b	140.10 c	139.93 b	141.33 c
Azo + Bac + Pseud	137.04 d	151.29 a	147.90 a	149.40 a	133.71 d
Standard error	1.09				
	**STY (t ha^−1^)**				
	**Rates of P_2_O_5_ (kg ha^−1^)**				
**Inoculation**	**0**	**45**	**90**	**135**	**180**
Control	167.30 a	153.13 b	155.33 b	167.25 b	194.90 a
Azo	174.20 a	196.68 a	185.48 a	131.25 b	198.55 a
Bac	163.15 a	182.98 a	179.10 a	194.90 a	165.43 a
Pseud	170.85 a	151.25 b	202.55 a	199.73 a	201.25 a
Azo + Bac	205.03 a	211.28 a	141.65 b	188.33 a	184.78 a
Azo + Pseud	168.08 a	185.53 a	203.30 a	201.28 a	187.08 a
Bac + Pseud	180.03 a	181.75 a	201.43 a	218.43 a	202.28 a
Azo + Bac + Pseud	173.88 a	158.25 b	172.03 b	147.95 b	185.68 a
Standard error			11.85		
	**SUY (t ha^−1^)**				
	**Rates of P_2_O_5_ (kg ha^−1^)**				
**Inoculation**	**0**	**45**	**90**	**135**	**180**
Control	24.45 a	21.99 b	22.14 b	23.85 b	26.80 a
Azo	25.16 a	30.29 a	26.28 a	18.23 b	27.10 a
Bac	21.75 a	24.69 b	24.69 b	27.51 a	24.13 a
Pseud	24.02 a	22.56 b	30.84 a	32.19 a	27.50 a
Azo + Bac	27.25 a	30.33 a	21.17 b	26.35 a	25.87 a
Azo + Pseud	25.08 a	26.27 b	28.11 a	28.56 a	27.61 a
Bac + Pseud	27.18 a	26.11 b	28.07 a	29.93 a	27.57 a
Azo + Bac + Pseud	23.51 a	24.14 b	24.98 b	21.03 b	24.76 a
Standard error	1.69				

Averages followed by the same letter in the columns belong to the same group by the Scott and Knott test, at 5% probability. Control (without inoculation); Azo (*Azospirillum brasilense*); Bac (*Bacillus subtilis*); Pseud (*Pseudomonas fluorescens*); Azo + Bac (*A. brasilense* + *B. subtilis*); Azo + Pseud (*A. brasilense* + *P. fluorescens*); Bac + Pseud (*B. subtilis* + *P. fluorescens*); Azo + Bac + Pseud (*A. brasilense* + *B. subtilis* + *P. fluorescens*).

**Figure A1:**
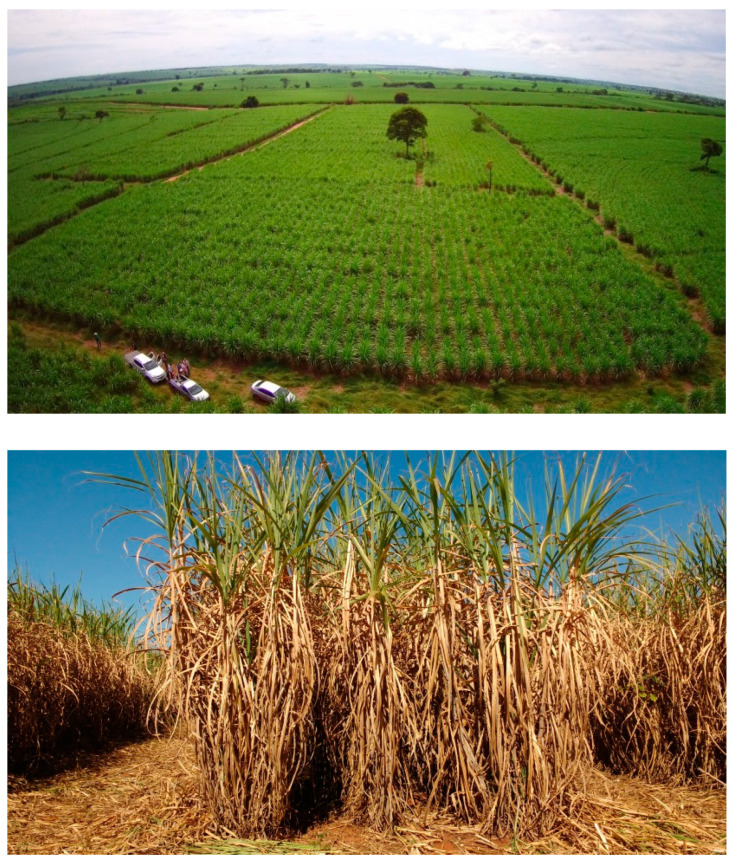

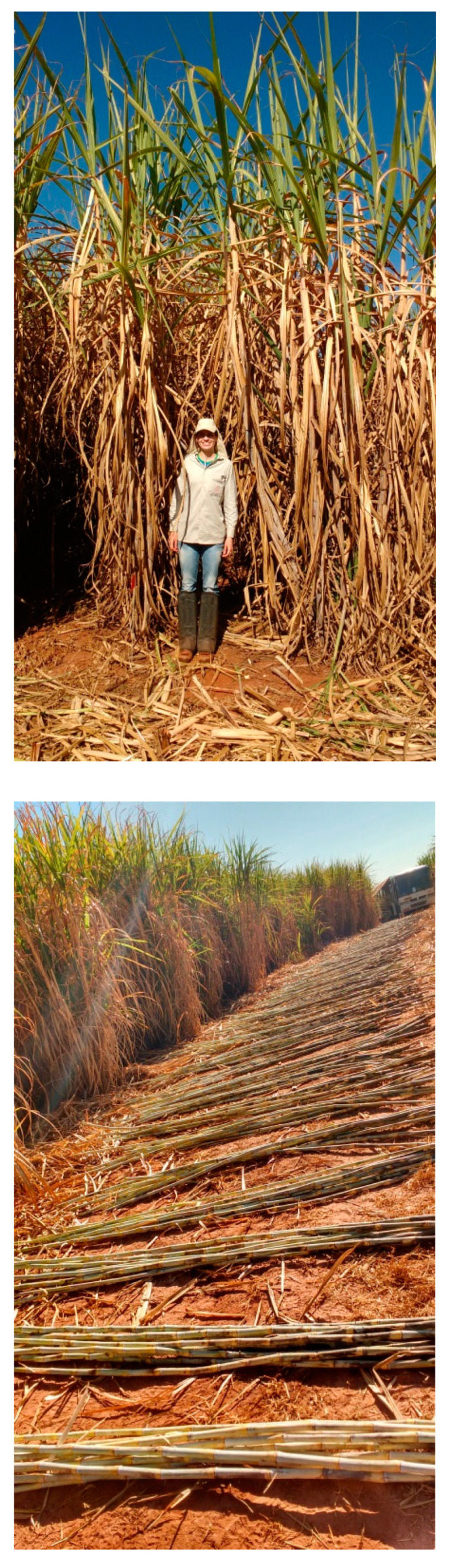
Photos of the experimental area with sugarcane plant samples for technological quality. Suzanapólis—SP, Brazil.

## Data Availability

All data generated or analyzed during this study are included in this published article.
